# Preoperative Nutrition in Bariatric Surgery: A Narrative Review on Enhancing Surgical Success and Patient Outcomes

**DOI:** 10.3390/nu17030566

**Published:** 2025-02-02

**Authors:** Daniel Simancas-Racines, Evelyn Frias-Toral, Martín Campuzano-Donoso, Daniel Ramos-Sarmiento, Raynier Zambrano-Villacres, Claudia Reytor-González, Luigi Schiavo

**Affiliations:** 1 Centro de Investigación en Salud Pública y Epidemiología Clínica (CISPEC), Facultad de Ciencias de la Salud Eugenio Espejo, Universidad UTE, Quito 170527, Ecuador; dsimancas@ute.edu.ec (D.S.-R.); martincd01@hotmail.com (M.C.-D.); danielrs2012@gmail.com (D.R.-S.); 2 Escuela de Medicina, Universidad Espíritu Santo, Samborondón 0901952, Ecuador; 3 Escuela de Nutrición y Dietética, Universidad Espíritu Santo, Samborondón 0901952, Ecuador; rzambranovillacres@uees.edu.ec; 4 Department of Medicine, Surgery and Dentostry “Scuola Medica Salernitana”, University of Salerno, 84081 Baronissi, Italy

**Keywords:** obesity, bariatric surgery, preoperative micronutrient deficiencies, micronutrient supplementation, healthcare

## Abstract

Bariatric surgery has become the preferred treatment for individuals with morbid obesity. Nutrition is key in optimizing surgical outcomes by reducing risks and enhancing recovery. Preoperative strategies, such as reducing body fat, decreasing liver size, and improving metabolic profiles, have been shown to facilitate safer surgical procedures with fewer complications. This narrative review aims to provide an analysis of the fundamental role of preoperative nutritional management in improving bariatric surgery outcomes, emphasizing the importance of addressing specific nutritional challenges to enhance surgical safety, recovery, and overall health. Preoperative nutritional interventions focus on correcting comorbidities and nutritional deficiencies, particularly hypovitaminosis and micronutrient imbalances, through a multidisciplinary approach involving nutritionists and other healthcare professionals. These interventions not only prepare patients for the physiological demands of surgery but also initiate a period of adaptation to new dietary habits, aiming to improve long-term compliance and mitigate risks such as postoperative weight regain and dumping syndrome. Adopting dietary changes, such as very low-calorie or ketogenic diets 6–12 weeks before surgery, enhances adherence to postoperative restrictions and overall surgical success. Future research should focus on developing comprehensive guidelines for preoperative nutritional care to improve patient outcomes globally.

## 1. Introduction

The global prevalence of obesity continues to rise at an alarming rate, with the World Health Organization estimating that more than 890 million adults were living with obesity in 2022, a number that has likely increased significantly since then [1]. Obesity is not only a major public health concern but also a driver of numerous comorbidities, including type 2 diabetes, cardiovascular disease, hypertension, and others, which collectively place a substantial burden on healthcare systems worldwide. For individuals with morbid obesity, bariatric surgery (BS) has emerged as the most effective and sustainable treatment for achieving significant weight loss and reducing the risk of obesity-related comorbidities [2,3,4,5].

The success of BS extends beyond the procedure itself. Evidence shows that appropriate and ongoing nutritional care, tailored to the type of surgery performed, significantly enhances its outcomes. Studies indicate that the combination of BS with preoperative and postoperative nutritional management not only promotes substantial initial weight loss but also supports long-term maintenance, often for two years or more [6,7]. Despite this, the importance of preoperative nutritional preparation is often underemphasized in existing clinical practice and the literature.

In recent years, the sharp rise in obesity prevalence has been accompanied by increasing public awareness and acceptance of BS, resulting in a notable rise in the number of surgeries performed globally. This surge has also brought to light the critical role of preoperative preparation, particularly in addressing the nutritional needs of bariatric patients [4,8]. Preoperative nutritional management plays a pivotal role in optimizing surgical outcomes by reducing surgical risks and enhancing postoperative recovery. It is especially crucial for high-risk patients, as strategies such as structured medical nutritional programs, preoperative weight reduction, and gastric balloon placement have been shown to modify body composition [9,10,11], decrease liver size [4,12,13], and improve metabolic profiles [14]. These interventions facilitate safer surgical procedures with fewer complications, highlighting the necessity of tailored preoperative care [7,15,16].

Vitamin and mineral deficiencies, particularly those involving vitamin D, iron, vitamin B12, and folate, are frequently observed in patients undergoing BS. These deficiencies are influenced by both the surgical alterations to the gastrointestinal anatomy and preexisting nutritional deficits, which can be further exacerbated by poor adherence to supplementation protocols [17,18]. If left unaddressed, these deficiencies can negatively impact surgical outcomes and overall patient health, increasing the risk of complications and hindering recovery [3,18]. Consequently, preoperative assessments, including biochemical testing, dietary evaluations, and targeted supplementation, have become integral components of BS preparation, ensuring patients enter surgery with optimized nutritional status [19,20].

Despite the growing recognition of preoperative nutrition’s role, there remains a significant gap in the literature regarding standardized approaches to address these challenges comprehensively. Current guidelines often lack specific recommendations for individualized care, particularly for addressing micronutrient and macronutrient deficiencies in diverse patient populations. This highlights the need for a multidisciplinary care model that integrates evidence-based dietary planning, targeted supplementation, and lifestyle counseling into preoperative protocols.

This narrative review aims to fill this gap by providing a detailed analysis of the critical role of preoperative nutritional management in enhancing the outcomes of BS. The review explores the challenges associated with micronutrient and macronutrient deficiencies, discusses practical strategies for nutritional assessment and intervention, and integrates current clinical guidelines. Additionally, it emphasizes the importance of a multidisciplinary and personalized care model while identifying opportunities for future research to advance preoperative nutritional strategies.

## 2. Preoperative Nutritional Deficiencies

### 2.1. Common Micronutrient Deficiencies

Patients undergoing BS often have poor nutritional status due to factors such as chronic illness, inadequate caloric intake, malabsorption, and increased energy demands [21,22]. These patients frequently exhibit micronutrient deficiencies even before surgery, with common deficits in vitamin D, folate, vitamin B12, and iron, among other ones [21,23,24]. Preoperative screening for these deficiencies is recommended to ensure timely interventions, as addressing them can prevent worsening conditions and can reduce the risk of postoperative complications [23,25].

Micronutrient deficiencies in bariatric patients result from both preexisting dietary habits and physiological changes induced by surgery. For instance, iron deficiency, particularly prevalent in procedures like the Roux-en-Y gastric bypass (RYGB), can lead to iron-deficiency anemia, causing fatigue, cognitive impairments, and immune dysfunction, which hinder wound healing and recovery. Vitamin D deficiency, often attributed to insufficient dietary intake, reduced absorption, and limited sun exposure, increases risks of osteoporosis, fractures, and infections, thereby prolonging postoperative recovery [26]. Similarly, vitamin B12 deficiency, resulting from gastrointestinal alterations, can cause megaloblastic anemia and neurological symptoms, while folate deficiency, often linked to impaired absorption, may contribute to anemia and complications during pregnancy. If left untreated, these deficiencies can lead to chronic conditions, such as osteoporosis and irreversible neurological damage, significantly increasing patient morbidity and impacting recovery [23].

A study by Ben-Porat et al. [23] analyzed nutritional deficiencies before and after sleeve gastrectomy (SG) in 77 patients over a 12-month follow-up. Prior to surgery, 99.4% of participants had vitamin D deficiencies, 40.9% had elevated Parathyroid Hormone (PTH) levels, and significant proportions had iron (47.1%), folate (32%), and vitamin B12 (13.1%) deficiencies. While anemia and vitamin B12 deficiencies worsened postoperatively (from 16.7% to 20%, *p* < 0.001), deficiencies in iron, folate, vitamin D, and PTH levels improved significantly. The study concluded that addressing preoperative deficiencies and tailoring supplementation programs based on routine blood tests could prevent postoperative nutritional deficits [23]. Similarly, Flancbaum et al. [27] reported comparable results, identifying vitamin D (68%), iron (43.9%), ferritin (8.4%), hemoglobin (22%), and thiamine (29%) as common preoperative deficiencies. Another study investigated the development of nutritional deficiencies following SG and highlighted a potential link between preoperative nutritional status and postoperative deficiencies. The findings emphasized that patients with preoperative deficiencies are particularly in need of careful monitoring and supplementation of micronutrients and protein after surgery [28]. These deficiencies, if not identified and managed effectively, can negatively impact surgical outcomes, increment complications, and delay recovery. Addressing these nutritional gaps is essential for optimizing patient health before surgery and improving long-term bariatric outcomes.

A cross-sectional study evaluated the prevalence of hypovitaminosis D in 206 patients scheduled for BS found that vitamin D insufficiency and deficiency were prevalent and associated with a higher body mass index (BMI) and the female sex [29]. Similar findings were reported in a study of 56 individuals with severe obesity (BMI > 35 kg/m^2^), where vitamin D deficiency correlated strongly with a higher BMI, the African-American race, and limited sunlight exposure [30]. Moreover, BS can exacerbate vitamin D deficiency in the postoperative period [31]. A systematic review highlights this association, linking hypovitaminosis D to various adverse outcomes after surgery [32]. Deficiency in vitamin D may lead to complications, such as muscle loss and reduced bone density. To mitigate these risks, it is essential to conduct preoperative vitamin D assessments and establish a rigorous postoperative follow-up plan, including vitamin D supplementation and regular monitoring of serum levels after all bariatric procedures [29].

To minimize these risks, bariatric patients should undergo thorough preoperative nutritional assessments. Interventions, such as vitamin supplementation and dietary modifications, should be implemented to address deficiencies before surgery. Continuous postoperative monitoring is crucial to sustain long-term success and improve patient outcomes [33].

### 2.2. Macronutrient Concerns

Contrary to the common perception of obesity as a state of overnutrition, patients with obesity who are candidates for BS often experience significant macronutrient deficiencies. These deficiencies are primarily attributed to poor dietary quality, characterized by the intake of high-calorie but nutrient-poor foods [34,35]. Additionally, factors such as sedentary lifestyles, altered metabolism, and chronic inflammation, further exacerbate the problem [36,37,38,39,40]. Addressing these deficits is crucial to optimizing surgical outcomes and minimizing postoperative complications, which underscores the importance of careful preoperative nutritional management.

Among macronutrients, proteins are especially vital due to their roles in tissue repair, immune function, and the preservation of lean body mass. In candidates for BS, ensuring adequate protein intake is critical, as these individuals are at increased risk of muscle loss resulting from preexisting dietary imbalances and the catabolic effects of surgery. When protein deficiency is present before surgery, it can impair wound healing, increase susceptibility to infections, and delay recovery from surgical stress [41]. Studies have reported varying prevalence rates of protein deficiencies in this population, with up to 10% of participants showing low levels of albumin and transferrin, while others report rates as high as 27% [34,42,43,44,45]. While biomarkers, such as albumin and transferrin, provide useful insights into protein status, they are not definitive measures [46]. Nonetheless, ensuring sufficient protein intake before surgery is a critical step in preserving lean body mass and supporting immune function, which are essential for successful recovery.

Despite the caloric excess often associated with obesity, dietary imbalances are common, with high intakes of fats and carbohydrates and insufficient consumption of protein-rich foods, such as lean meats, fish, dairy, and legumes [47]. This dietary imbalance contributes to muscle loss, weakened immune function, and delayed healing, all of which heighten the risk of postoperative complications. Furthermore, BS itself introduces unique barriers to maintaining adequate protein intake. Anatomical changes resulting from procedures such as RYGB or SG significantly reduce stomach capacity and alter gastrointestinal function, making it challenging for patients to consume sufficient protein [47]. Postoperative protein intolerance further complicates this, as changes in bile and pancreatic enzyme secretion, combined with a reduced stomach size, can cause gastrointestinal discomfort, nausea, and vomiting when consuming protein-rich foods [48]. This intolerance exacerbates the risk of protein deficiency and protein-energy malnutrition, which may manifest as muscle loss, weakness, delayed wound healing, and compromised immune function [49].

Preoperative nutritional counseling is essential to addressing these challenges. Counseling helps patients understand the importance of adequate protein intake and equips them with strategies to meet their nutritional requirements both before and after surgery [50,51]. For many, this includes incorporating protein supplements, such as protein shakes or liquid protein sources, to bridge the gap between dietary intake and protein needs. In addition, patients should be educated on protein sources that are better tolerated after surgery, such as lean meats, fish, eggs, and legumes, and should be encouraged to consume smaller, more frequent meals to minimize gastrointestinal discomfort and maximize protein intake [52]. These tailored dietary strategies not only help patients meet preoperative protein requirements but also provide a foundation for managing nutritional needs in the postoperative period.

Optimizing macronutrient intake, with a particular emphasis on protein, is essential for improving outcomes in BS patients. The unique challenges faced by individuals with obesity, from inadequate dietary patterns to post-surgical anatomical and physiological changes, necessitate targeted nutritional interventions. By addressing these challenges through tailored strategies, healthcare providers can significantly enhance surgical success and support long-term recovery.

### 2.3. Assessment of Nutritional Status

Perioperative nutritional support involves providing nutrition via oral (including diets or oral nutritional supplements), enteral, or parenteral methods. This approach has demonstrated significant benefits, such as reducing complications, shortening hospital stays, decreasing morbidity and mortality, and lowering healthcare costs. Optimizing nutritional status before elective surgery is crucial for achieving better postoperative outcomes and facilitating recovery [20,53]. Preoperative nutritional evaluation is critical for patients undergoing weight-loss surgery, as many present with at least one vitamin or mineral deficiency [54]. It is estimated that up to two-thirds of these patients are malnourished before surgery, a risk factor often underestimated [55]. Screening is vital because malnutrition, primarily due to reduced food intake, can significantly impact surgical outcomes [14,20,53]. However, despite its importance, routine preoperative micronutrient screening is not yet a standard practice in many weight-loss surgery programs [21].

The American Society for Metabolic and Bariatric Surgery Integrated Health Nutritional Guidelines recommend comprehensive preoperative screenings for nutrients such as thiamine, vitamin B12, folic acid, iron, vitamin D, calcium, fat-soluble vitamins (A, E, K), zinc, and copper. These screenings are especially critical for patients undergoing an RYGB or biliopancreatic diversion/duodenal switch, though caution is advised in interpreting certain test results [54].

The European Society for Clinical Nutrition and Metabolism (ESPEN) Good Clinical Practice guidelines advise perioperative nutritional support for malnourished patients or those at risk of nutritional deficiencies, as well as for patients expected to have minimal oral intake for extended periods [20]. Effective nutritional assessment should predict clinical outcomes, be low-cost, and be quickly administered [53]. Comprehensive evaluations typically include assessments of dietary intake, nutritional requirements, functional status, and body composition, using anthropometric and laboratory parameters. Common tools include the Subjective Global Assessment, Nutritional Risk Index, and measurements like BMI, mid-arm circumference, and serum albumin levels [22].

Preoperative nutritional assessment for BS often involves a range of biochemical markers to evaluate the patient’s nutritional status [56]. Key markers include serum albumin and prealbumin, which reflect protein status, with low levels suggesting malnutrition [57]. Additional markers, such as C-reactive protein to assess inflammation [58] and hemoglobin to detect anemia (often due to deficiencies in iron or vitamin B12), are also essential [59]. Iron and ferritin levels assess iron status, while vitamin D, B12, and folate levels are crucial for evaluating potential deficiencies that are common in bariatric patients [57]. Furthermore, total lymphocyte count and transferrin levels can indicate immune function and protein status [60]. These markers are essential for identifying nutritional issues early, enabling appropriate interventions that enhance patient recovery and surgical outcomes.

Albumin, the most abundant protein in human serum, has long been used as an indicator of malnutrition in clinically stable patients [57]. However, a 2015 meta-analysis highlighted that hypoalbuminemia is not a normal consequence of aging and requires careful evaluation to identify underlying causes, including, but not limited to, nutritional deficits [61]. While severe hypoalbuminemia correlates with extended intensive care unit (ICU) stays, prolonged postoperative recovery, and increased complications, these complications themselves are the strongest predictors of resource use, such as ICU time [62]. Others agree that it is not solely indicative of malnutrition and requires further assessment to determine underlying causes, particularly in elderly patients [63]. Transferrin, a transport protein for iron with a half-life of approximately 10 days, has also been used as a nutritional marker [64,65]. However, its reliability is limited due to its dependence on iron status, with levels decreasing in severe malnutrition but increasing in cases of iron deficiency, which complicates its interpretation. Consequently, its use for nutritional assessment is no longer recommended [63,66]. Prealbumin, or transthyretin, has emerged as a more effective marker for detecting acute nutritional changes due to its shorter half-life of two to three days [57]. This protein, synthesized by the liver and confined to the intravascular space, meets key criteria for an ideal biomarker, making it particularly useful for stratifying patients by risk of complications and outcomes [67]. Recent algorithms incorporating prealbumin have been developed to assess nutritional risk in both general medical and intensive care settings [63]. Other laboratory markers other than the visceral proteins discussed in this review can be found in Table 1 [57].

**Figure 1:**
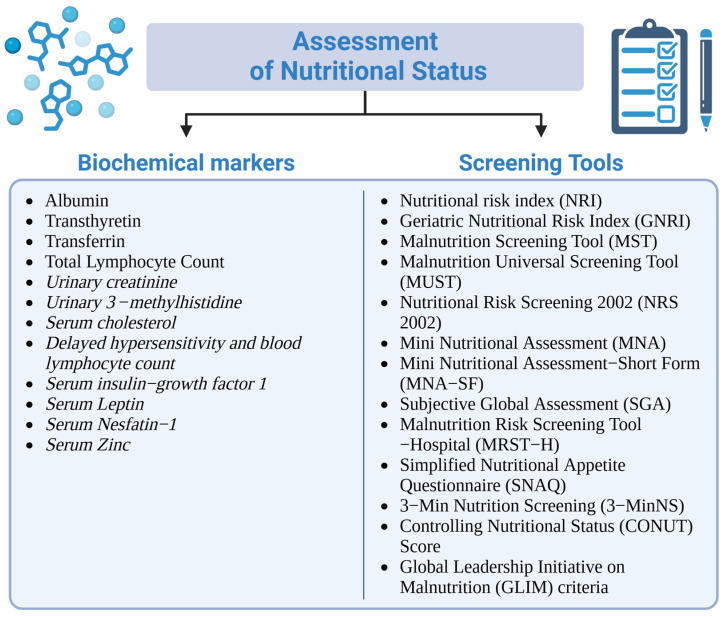
Options to assess nutritional status in patients: To assess nutritional status, it is essential to combine the analysis of biochemical markers (such as albumin, transferrin, and serum zinc) with the use of screening tools, such as the Mini Nutritional Assessment (MNA) and Global Leadership Initiative on Malnutrition (GLIM) criteria. This combined approach enables the identification of malnutrition risks and facilitates targeted clinical interventions [20,23,60,61,62,63,64,65,66,67].

Despite the availability of over 70 nutritional screening tools, determining the degree of malnutrition remains complex. These tools range from simple evaluations of appetite and weight loss to more detailed assessments involving anthropometric and biochemical data [20,53,100]. While classical indicators like body weight, BMI, and serum albumin are still widely used, modern approaches highlight their limitations, particularly the sensitivity of serum albumin to inflammation and its long half-life [101]. Incorporating routine and comprehensive preoperative nutritional assessments is vital for optimizing outcomes in weight-loss surgery patients. Addressing malnutrition before surgery ensures improved recovery, reduces complications, and enhances long-term health outcomes [20,23,53]. Although numerous screening tools for detecting malnutrition are currently in use (Figure 1), none have been definitively validated. Further research is needed to establish their clinical reliability and effectiveness [20,102].

#### 2.3.1. Nutritional Risk Index (NRI)

The NRI is a valuable tool for assessing nutritional risk in hospitalized patients, particularly for predicting postoperative complications. It is calculated using serum albumin levels and the ratio of current to usual body weight with the formula:

NRI = 1.519 × Albumin (g/L) + [41.7 × (current weight/usual weight)]

The scores are interpreted as follows: >100 indicates a well-nourished status, 97.5–100 signifies mild malnutrition, 83.5–97.5 indicates moderate malnutrition, and <83.5 represents severe malnutrition [103]. Research highlights the NRI’s sensitivity, specificity, and positive predictive value for identifying patients at risk of surgical complications [104]. 

A retrospective study by Thieme et al. [22] evaluated the relationship between nutritional status and postoperative outcomes in 125 patients undergoing digestive system or abdominal wall surgery. Postoperative complications occurred in 50.4% of patients, with 26.4% experiencing infectious and 24% non-infectious complications. Nutritional assessments revealed that 65.6% were malnourished according to the Subjective Global Assessment, while 88.0% were classified as malnourished using the NRI. Severe malnutrition was identified in 17.6% of patients via SGA and 42.4% via NRI. Notably, a low NRI score was significantly associated with non-infectious complications (*p* = 0.0016) but not with infectious complications, reoperation, or 30-day mortality rates. These findings highlight the NRI and serum albumin levels as effective predictors of non-infectious postoperative complications, particularly in patients with malignant conditions [22]. Another observational study assessed the predictive value of the NRI and BMI for postoperative outcomes in 134 patients undergoing gastrointestinal surgery. The NRI identified a higher proportion of patients at nutritional risk (72.38%) compared to the BMI (28.35%) and showed significant correlations with postoperative complications, particularly in severely malnourished patients (NRI < 83.5, *p* < 0.006). The NRI was also significantly associated with postoperative wound infections and extended hospital stays, whereas the BMI showed no significant associations with complications. These results highlight the NRI as a more effective tool than the BMI for predicting postoperative morbidity and complications [22].

#### 2.3.2. Geriatric Nutritional Risk Index (GNRI)

The GNRI, introduced by Bouillanne et al. [105], is a tool designed to assess nutritional risk and predict malnutrition severity and mortality in hospitalized elderly patients. Its calculation is based on the formula:

GNRI = 1.489 × albumin (g/L) + 41.7 × (present weight/ideal weight) = 1.489 × albumin (g/L) + 41.7 × (BMI/22)

Based on the score, patients are classified as no risk (GNRI > 98), low risk (GNRI 92–98), moderate risk (GNRI 82–92), or major risk (GNRI < 82) [105].

A study investigating the relationship between preoperative GNRI and postoperative outcomes in elderly patients (>75 years) undergoing curative gastrectomy for gastric cancer analyzed 348 participants. Patients with a low GNRI (<92) had significantly higher rates of postoperative complications (26.0% vs. 15.3%, *p* = 0.013) compared to those with a high GNRI. Particularly, extra-surgical complications, such as pneumonia (*p* = 0.013), were more common in the low-GNRI group. These findings indicate that preoperative GNRI is an independent predictor of postoperative complications and may help identify high-risk elderly patients undergoing gastrectomy [102].

#### 2.3.3. Malnutrition Screening Tool (MST)

The MST is a simple, quick, and reliable screening method designed to identify patients at nutritional risk. It involves questions about appetite, nutritional intake, and recent weight loss. The MST has a total score of 7, with a score of 2 or higher indicating the need for further nutritional assessment and/or intervention [106]. The MST’s simplicity is one of its key strengths, it requires less than five minutes to complete, involves no complex calculations, and directly informs a care plan based on the final score [20]. A score of ≥2 signals the need for additional evaluation, making MST an efficient method for identifying patients at nutritional risk and guiding appropriate interventions [107].

#### 2.3.4. Malnutrition Universal Screening Tool (MUST)

The MUST is designed to assess nutritional risk in adult patients by evaluating three key factors: BMI, percentage of unintentional weight loss over the past six months, and the impact of illness on nutritional intake. The score categorizes patients as medium risk (score of 1) or high risk (score ≥ 2) [108]. Although MUST consists of only three questions, it requires calculations for BMI and recent weight loss percentages to determine the risk level accurately [107].

A study assessing the validity of the MST and the MUST against GLIM criteria analyzed data from 5270 hospitalized patients. The MST demonstrated higher accuracy, with a specificity of 89.9% (k = 0.591, *p* < 0.001). Both tools were associated with prolonged hospital stays and increased mortality, with the MST emphasizing unintentional weight loss and the MUST focusing on reduced food intake. It is recommended that healthcare professionals use either tool within the first 24–72 h of admission to identify and address nutritional risk effectively [107]. Another study evaluated the association between MUST scores, body composition, systemic inflammatory responses (SIR), and clinical outcomes in 363 patients undergoing colorectal cancer surgery. Of the patients, 79% had a MUST score of 0 (low risk), 9% scored 1 (medium risk), and 12% scored ≥2 (high risk). Higher MUST scores were significantly associated with prolonged hospital stays (78% of patients with a score of 2 had stays >7 days vs. 49% with a score of 0, *p* = 0.002) and lower 3-year survival rates (33% mortality for a score of 2 vs. 17% for a score of 0, *p* = 0.001). The MUST also correlated with phenotypes such as low skeletal muscle mass, adiposity, and SIR, making it a valuable tool for characterizing malnutrition and predicting clinical outcomes in colorectal cancer patients [109]. Similarly, in the context of BS, the MUST can be a valuable tool for assessing preoperative nutritional status, identifying patients at risk for complications, and guiding targeted interventions to improve postoperative recovery and long-term health outcomes.

#### 2.3.5. Nutritional Risk Screening 2002 (NRS 2002)

The NRS 2002 tool aims to identify undernourished patients who would benefit from nutritional intervention [110]. It consists of a preliminary phase with four questions: BMI < 20.5, recent weight loss, reduced food intake over the past week, and presence of a serious illness. If any of these factors apply, the patient proceeds to the screening phase, which evaluates weight loss, BMI, food intake, and disease severity (including major surgeries, cerebrovascular events, traumatic brain injuries, and bone marrow transplants). Each factor is scored between 0 and 3, and an additional point is added for patients over 70 years old [110,111]. A total NRS 2002 score of <3 indicates no nutritional risk, while a score ≥ 3 signals a high risk of malnutrition, necessitating nutritional support. The maximum possible score is 7. Validated in numerous studies, including randomized controlled trials, the NRS 2002 is recommended by the ESPEN for screening hospitalized patients [112].

#### 2.3.6. Mini Nutritional Assessment-Short Form (MNA-SF)

The MNA-SF is a condensed version of the original MNA, consisting of 6 items instead of 18. It assesses factors such as food intake, weight loss, mobility, psychological stress, neuropsychological issues, and BMI, yielding a maximum score of 14. Scores are categorized as follows: a score of 12–14 indicates normal nutritional status, 8–11 suggests a risk of malnutrition, and 0–7 signifies malnutrition [112]. While the full MNA provides a comprehensive assessment and correlates well with clinical evaluations and serum albumin levels, its length limits its practicality for routine screenings. The MNA-SF addresses this issue by offering a shorter, yet effective, alternative [113]. A study investigated the utility of the MNA-SF in identifying frailty in older adults, as defined by Fried’s criteria. The study included 1003 outpatients aged 65 or older and found that the MNA-SF had good sensitivity (71.2%) and specificity (92.8%) for frailty detection, with an area under the curve of 0.906. These results suggest that the MNA-SF is a valuable tool for screening frailty in older adults [114].

#### 2.3.7. Subjective Global Assessment (SGA)

The SGA evaluates both medical history (including food intake, weight loss, symptoms affecting oral intake, and functional capacity) and physical examination (such as loss of body fat, muscle mass, edema, and ascites). Patients are classified into three categories based on these assessments: grade A (well-nourished), grade B (mild/moderate malnutrition), and grade C (severely malnourished) [111]. The SGA is widely recognized as a reliable and valid method for assessing the nutritional status of hospitalized surgical patients [115].

Pham et al. [116] aimed to assess nutritional status using the SGA in patients undergoing abdominal surgery and to determine the incidence of malnutrition and its correlation with infectious complications. The study of 438 patients found that malnutrition was highly prevalent, with those classified as severely malnourished (SGA class C) having a higher rate of infectious complications compared to those in classes A and B. The study also highlighted that SGA, involving detailed history and physical examination, is a feasible and effective method for assessing nutritional status in surgical patients in this region [116]. The recent literature supports the SGA as a valid tool for nutritional assessment in both surgical and clinical settings. However, tools such as NRS 2002 may be equally or more effective in detecting nutritional issues associated with poor clinical outcomes, particularly in elderly hospitalized patients [117].

A systematic review of 111 studies involving 52,911 participants evaluated biomarkers for assessing malnutrition severity using established tools like MNA, SGA, and NRS 2002. While BMI, hemoglobin, and total cholesterol were identified as reliable markers of malnutrition in older adults, albumin and prealbumin were less predictive in acute illnesses contexts, reflecting inflammation rather than nutritional status [66]. Nutritional assessment is crucial before BS to ensure optimal outcomes and identify underlying deficiencies. According to the American Society for Parenteral and Enteral Nutrition, the primary goals of nutritional assessment are to document key nutritional parameters, identify risk factors and deficiencies, assess nutritional needs, and address medical, psychosocial, and socioeconomic factors influencing nutritional support [118]. The ESPEN emphasizes that nutritional assessment serves as the foundation for diagnosing malnutrition, considering clinical, psychological, social, and nutritional history, along with physical examination findings, such as weight, height, BMI, body composition, biochemical markers, and nutrient requirements [119]. Over time, various methods for nutritional assessment have been developed, ranging from complex and costly research-based techniques to more practical and affordable tools for routine clinical practice. The ideal assessment method must be both sensitive and specific, enabling accurate nutritional diagnoses, predicting outcomes, and monitoring nutritional interventions [110]. While the screening tools discussed in this review may be useful for assessing nutritional status prior to BS, there is a lack of studies specifically correlating these tools with outcomes in this population. Hence, there is a need for additional studies to develop definitive guidelines. A comprehensive approach to evaluating nutritional status before BS should include both clinical signs and biochemical markers of malnutrition, tailored to the clinical setting and available resources, to ensure effective nutritional support and optimize surgical outcomes.

## 3. Nutritional Interventions

### 3.1. Preoperative Dietary Plans

The preoperative phase of BS is a critical window for preparing patients to meet the metabolic and anatomical demands of the procedure. Among the key strategies employed, low-calorie diets and very low-calorie ketogenic diets (VLCKD) stand out as effective interventions for reducing liver size and improving surgical outcomes [120]. These dietary approaches not only facilitate weight loss but also optimize the metabolic environment, enhancing patient safety and long-term success.

VLCKD has gained significant attention for its rapid and profound effects on weight and liver volume reduction. Schiavo et al. [121] conducted a prospective pilot study involving a four-week ketogenic, micronutrient-enriched diet administered to 27 individuals with morbid obesity. The study reported a 10.3% weight loss in males and 8.2% in females, alongside a notable 19.8% reduction in left hepatic lobe volume. Furthermore, the intervention addressed preexisting micronutrient deficiencies, positioning patients for improved postoperative outcomes. Compliance rates were high, with no significant adverse effects observed, highlighting the feasibility and practicality of this approach [121].

Similarly, Pilone et al. [122] demonstrated the benefits of a standardized 30-day VLCKD protocol in 119 patients. The study revealed significant reductions in body weight, visceral fat, and liver volume, achieving a mean liver size reduction of 30%. Beyond these physical changes, the diet improved glycemic and lipid profiles, as reported by other authors [123], highlighting its systemic benefits. Importantly, patients reported satisfaction with the dietary regimen, and adverse effects were limited to mild and transient symptoms [122].

Barrea et al. [51] provided a broader perspective, highlighting VLCKD as an essential preoperative tool for bariatric patients. Their review emphasized the diet’s ability to reduce visceral adipose tissue and liver steatosis, facilitating laparoscopic surgery. They also underscored the high compliance rates associated with ketosis, which diminishes hunger and promotes adherence. This effect of metabolic benefits coupled with practical advantages positions VLCKD, now referred to as Very Low Energy Ketogenic Therapy (VLEKT), as a cornerstone of preoperative nutritional interventions [51,124].

The macronutrient composition of these diets is carefully designed to preserve lean body mass while achieving rapid weight loss. Typical VLEKT protocols restrict carbohydrates to 30–50 g/day, provide protein at 1.2–1.5 g/kg ideal body weight/day, and derive 30–40% of total energy from fats. These specifications are essential not only for efficacy but also for minimizing potential complications during the preoperative phase [122].

In cases where adherence to conventional diets proves challenging, alternative strategies have emerged. For example, Castaldo et al. [125] explored the use of enteral protein nutritional therapy, demonstrating its effectiveness in improving glycemic and lipid profiles in just four weeks. This approach underscores the need for flexible, individualized preoperative plans to meet diverse patient needs and preferences [125].

In conclusion, preoperative dietary strategies, such as VLEKT, play a pivotal role in preparing BS candidates. By reducing liver size, improving metabolic parameters, and addressing nutritional deficiencies, these interventions lay a solid foundation for safe surgeries and better long-term outcomes. Tailored approaches that account for individual preferences and challenges can further enhance these benefits, ensuring that patients are optimally prepared for their transformative journey.

### 3.2. Micronutrient Supplementation

Micronutrient supplementation is a critical component of preoperative preparation for BS, complementing dietary interventions to address common nutritional deficiencies in this population. Despite caloric excess, obesity is frequently associated with micronutrient deficiencies due to poor dietary quality and altered metabolism. Preoperative correction of these deficiencies is essential to minimize surgical risks and optimize recovery (Table 2).

Among the most prevalent deficiencies is vitamin D, affecting over 66% of patients, as highlighted by several authors [26,126]. This deficiency compromises bone health and immune function, making its correction a priority. High-dose cholecalciferol supplementation (4000–6000 IU/day) is recommended for severe deficiency, transitioning to maintenance doses of 2000 IU/day post-surgery to sustain adequate levels [127].

Iron deficiency, often accompanied by anemia, is another common issue. The British Obesity and Metabolic Surgery Society guidelines recommend daily iron supplementation (100–200 mg elemental iron) to restore levels, with vitamin C co-administration to enhance absorption. For patients who are intolerant to oral formulations, intravenous iron provides an effective alternative [128].

Vitamin B12 and folate deficiencies are also critical to address. Vitamin B12 deficiency, often resulting from impaired absorption, typically requires intramuscular injections of 1000 µg every two weeks or high-dose oral supplements to restore levels. Folate supplementation at 2 mg/day is effective for addressing deficiencies, particularly in patients with anemia [126].

Timing is critical in micronutrient interventions. Initiating supplementation at least 10 weeks prior to surgery allows sufficient time for deficiencies to be corrected. Regular monitoring and dose adjustments ensure optimal levels, supporting the body’s resilience to surgical and postoperative challenges.

As patients adapt to these dietary and nutritional changes, another layer of preparation focuses on addressing behavioral and lifestyle factors that underpin long-term adherence and success.

### 3.3. Behavioral and Lifestyle Modifications

Integrating behavioral counseling into preoperative preparation bridges the gap between nutritional planning and patient compliance. A multidisciplinary approach, including psychological readiness, is essential for sustained lifestyle changes and maximizing the benefits of BS. Structured behavioral interventions, such as Cognitive-Behavioral Therapy, have proven effective in modifying maladaptive eating behaviors and promoting adherence to dietary plans. Schiavo et al. [120] highlighted that Cognitive-Behavioral Therapy not only reduced emotional eating but also improved overall dietary compliance, addressing a common barrier to surgical readiness.

Mindfulness-based approaches and motivational interviewing further enhance engagement by fostering self-awareness and intrinsic motivation. These techniques are particularly helpful in managing eating disorders, such as binge eating or night eating syndrome, which are prevalent among bariatric candidates [126].

Addressing psychological comorbidities, such as anxiety and depression, is equally important. Untreated psychological conditions can impair adherence to preoperative regimens and increase the likelihood of postoperative complications. By integrating psychological support into the care pathway, patients are better prepared to meet the demands of surgery and adapt to the lifestyle changes it requires.

Behavioral counseling also focuses on setting realistic goals and creating accountability frameworks. Collaborative goal setting helps patients align their expectations with achievable milestones, fostering a sense of accomplishment and reinforcing motivation throughout the preoperative phase.

**Figure 2:**
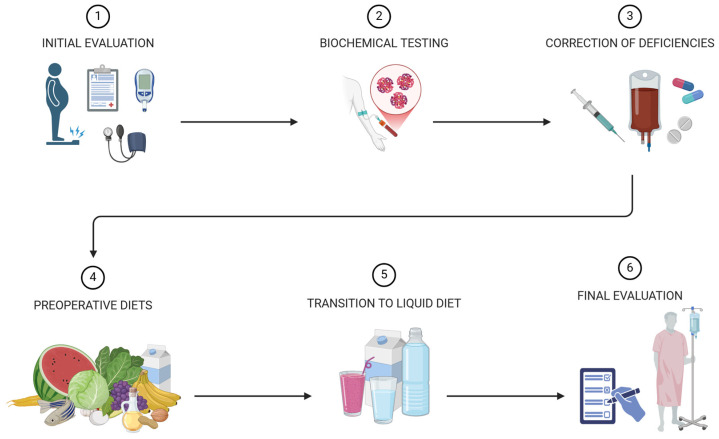
Preoperative preparation and nutritional interventions in bariatric surgery. The preoperative preparation for BS includes six stages: initial evaluation of medical and dietary history to identify comorbidities and deficiencies; biochemical testing to assess levels of vitamin D, iron, B12, folate, proteins, and other micronutrients; correction of deficiencies with tailored supplementation; implementation of preoperative diets such as VLEKT to reduce liver size and weight; transition to a liquid diet 24 h before surgery with clear liquids and protein shakes; and a final review to confirm nutritional correction and psychological readiness [5,119,120,126].

Preoperative preparation for BS is a multifaceted and dynamic process that harmonizes dietary interventions, micronutrient optimization, and behavioral counseling. These elements are interconnected, each reinforcing the others to create a comprehensive strategy. Together, they improve surgical safety and build a foundation for long-term health improvements, ensuring that patients are fully equipped for success on their surgical journey (Figure 2).

## 4. Clinical Guidelines and Best Practices

### 4.1. Overview of Existing Guidelines

Healthcare providers often offer patients undergoing BS varied and sometimes conflicting nutritional information for the preoperative and postoperative phases. A variety of educational guidelines and protocols exist to prepare patients for surgery, aiming to optimize outcomes and reduce complications [2,5,43,44,129]. Key time segments of preoperative nutrition are described below:

#### 4.1.1. The Initial Phase

As a consequence of the first nutritional consultation, the patient is advised to start implementing a bariatric diet, typically two weeks to six months before surgery. This diet includes recommendations to reduce fat and sugar intake, increase protein consumption, take micronutrient supplements, and lower carbohydrate intake [4,120,129]. Early adoption of these dietary changes increases the likelihood of adherence to postoperative dietary restrictions.

Adhering to preoperative dietary guidelines can significantly decrease the likelihood of experiencing slow weight loss, weight regain, weight plateaus, dehydration, discomfort, abdominal pain, indigestion, heartburn, and dumping syndrome. The patient’s success is influenced by several factors that impact their ability to follow the recommended protocols [130]. These include financial considerations, such as the capacity to purchase protein powder, vitamin, and micronutrient supplements, as well as fresh fruits and vegetables. Social factors also play a role, which involve home life, family support, and environmental influences. Additionally, internal psychological factors, such as personal feelings of self-worth or dissatisfaction, further impact adherence to these protocols [5,129,131,132].

Healthcare providers are encouraged to guide patients through the stages of dietary progression prior to surgery. These stages include transitioning from clear liquids to full liquids, then to soft foods, and eventually to regular-textured foods. Practicing these transitions helps patients acclimate to the food options required during recovery and allows providers to evaluate patients’ attitudes toward food and the prescribed diet. Common emotional responses may include frustration, grief over losing familiar comfort foods, or fear of failing to meet weight loss and lifestyle goals. Comprehensive education minimizes these negative outcomes, reduces the risk of weight regain, and equips patients to adapt to the postoperative plan and associated psychological adjustments [45,128,129].

#### 4.1.2. Preoperative Period

Patients are required to follow a low-fat diet beginning at least two weeks prior to surgery, as recommended by the VLEKT guidelines. The primary objectives of this preoperative dietary intervention are to facilitate initial weight loss and, most importantly, to reduce the liver size. A smaller liver is critical in bariatric surgery, as it enhances the surgeon’s access the stomach by minimizing obstructions in the operative field. This not only shortens the duration of the procedure but also significantly reduces the risk of intraoperative complications, such as liver injury or bleeding [4,8,9,10,11,12,13,17,120]. The diet typically includes a high-protein meal replacement shake, carefully selected and approved by a dietitian, to be consumed two to three times per day. Additional components include a portion of raw vegetables, a serving of fruit for snacking, and a small, calorie-controlled meal of no more than 400 calories. While patients may begin this dietary regimen at any point before surgery, it must start no later than two weeks before the scheduled surgery date [4,8,9,10,11,12].

Patients are advised to follow a full liquid diet during the 24 h before surgery. This diet permits unlimited consumption of calorie-free beverages or those containing no more than 15 g of sugar per serving. Additionally, it includes protein shakes, milk, yogurt, unsweetened applesauce, broth, gelatin, and pureed soups. To ensure the highest level of surgical safety, patients must cease all food and liquid intake after midnight. [17,51,120,128].

### 4.2. Multidisciplinary Approach

A multidisciplinary approach to bariatric care significantly enhances weight loss outcomes and ensures long-term sustainability. In addition to the surgeon, the care team should include a primary care physician to screen for and manage underlying medical conditions, and an endocrinologist or internist experienced in obesity management to optimize the patient for surgery by addressing comorbidities with medications and other treatments. A registered dietitian plays a crucial role in providing preoperative nutritional education and guidance on dietary adjustments, while a clinical psychologist evaluates mood, cognitive function, psychosocial status, substance use, social and family support, and the patient’s motivation and readiness to adopt behavioral changes. An exercise physiologist is also essential, assisting in the development of a regular exercise program tailored to the patient’s needs. This comprehensive approach addresses the physical, psychological, and behavioral aspects of care, ensuring patients are well-prepared for surgery and the postoperative lifestyle changes necessary for success [23,133,134].

As described by Schiavo et al. [131] a preoperative low-calorie ketogenic diet may not only reduce liver volume and visceral fat but also influence surgical outcomes, drainage production, postoperative hemoglobin levels, and hospital stays. Similarly, Patel et al. [135], conducted a matched cohort study comparing weight loss outcomes in individuals with obesity who participated in a preoperative multidisciplinary program (including a nutritionist, physician, and fitness trainer) with those who underwent surgery without such preparation. Patients who received multidisciplinary care before RYGB achieved significantly greater weight loss at 6 and 12 months post-surgery compared to those without the program. Delgado-Floody et al. [136] analyzed postoperative outcomes in 21 individuals who underwent a 4-month preoperative program that involved physical exercise, nutritional counseling, and education, three times per week. The study reported significant improvements in preoperative conditions and reduced risks of morbidity and mortality, underscoring the value of multidisciplinary preoperative care.

### 4.3. Individualized Care

Recent studies indicate that patients with severe obesity often experience micronutrient deficiencies at a higher rate compared to individuals of normal weight. Approximately 20–30% of candidates for BS present with micronutrient deficiencies before the procedure [2,3,4,137]. These deficiencies are often linked to factors such as the consumption of calorie-dense but nutrient-poor foods, reduced bioavailability of nutrients like vitamin D, chronic inflammation [138] that interferes with iron metabolism, and small intestinal bacterial overgrowth [139], which can result in deficiencies in thiamine, vitamin B12, and fat-soluble vitamins. Despite these challenges, the benefits of BS, particularly the resolution or significant improvement of chronic conditions like diabetes, hypertension, and hyperlipidemia, greatly outweigh the potential complications associated with preoperative nutrient deficiencies [20,137].

Nutritional needs and challenges play a critical role in guiding patient management throughout the surgical process. Over the years, both the number of BS procedures and confidence in specific techniques have increased. SG has become the most commonly performed operation, surpassing RYGB. Several factors contribute to this preference: SG avoids anastomosis, mesenteric defects, and malabsorption-related complications. Additionally, it is associated with a lower incidence of dumping syndrome, allows for endoscopic access to the stomach, and involves a less complex surgical technique compared to RYGB. These advantages make SG an attractive option for both patients and surgeons [137,140,141].

These considerations have shaped a structured approach to the patient’s clinical journey, divided into several key stages. The preoperative stage focuses on achieving weight loss before surgery to minimize surgical risks and optimize patient readiness. The postoperative stage aims to sustain long-term weight loss, address nutritional needs, and manage associated comorbidities effectively. Enhanced Recovery After Surgery (ERAS) protocols are designed to promote smoother perioperative recovery through evidence-based practices, emphasizing the importance of ongoing multidisciplinary follow-up to ensure sustained success and patient well-being. This staged approach ensures comprehensive care, addressing both immediate and long-term goals for BS patients [5,137].

Although this review provides an extensive synthesis of current evidence on preoperative nutritional management, its narrative nature limits the methodological rigor compared to systematic reviews. The selection and inclusion of studies were conducted comprehensively; however, the potential for selection bias and the absence of quantitative analysis must be acknowledged. Furthermore, the lack of consensus in clinical practices and variability in patient adherence highlight the need for future research to establish standardized, evidence-based guidelines that address the diverse needs of bariatric patients globally.

## 5. Conclusions

Candidates for BS often consume unbalanced diets rich in refined foods, added sugars, and fats, which exacerbate nutritional deficiencies and deteriorate overall health. Common deficiencies, such as hypovitaminosis and insufficient levels of folic acid, zinc, calcium, and iron, are further compounded by medications prescribed for comorbidities. These deficiencies pose significant surgical and nutritional challenges during both the preoperative and postoperative periods. Therefore, it is essential to identify and correct these deficiencies early, with the support of a nutritionist and a multidisciplinary team to comprehensively address each stage of care.

Adhering to preoperative preparation guidelines, low-calorie or ketogenic diets, such as Very Low Energy Ketogenic Therapy (VLEKT), are beneficial when implemented between six months and two weeks before surgery. Early adoption of these dietary changes facilitates adaptation to new eating habits, addresses nutritional deficiencies, manages existing comorbidities, and reduces the likelihood of complications, such as treatment refusal, weight regain, and postoperative dumping syndrome. Patients who adopt these dietary habits earlier tend to demonstrate better compliance with postoperative dietary restrictions, leading to greater long-term success.

As preoperative nutritional interventions become increasingly routine, research is needed to define standardized nutritional protocols, particularly for addressing nutritional deficiencies. For example, while reducing weight before surgery to minimize complications remains a topic of debate, most surgeons agree that reducing visceral fat and liver size facilitates the procedure and lowers surgical risks. Although the choice of diet varies, VLEKT has demonstrated effectiveness in promoting rapid weight loss, making it a viable recommendation for many patients.

To summarize, optimizing preoperative nutritional management is critical for enhancing the success of bariatric surgery and improving patient outcomes. While the evidence supports early dietary interventions and the correction of deficiencies as essential components of care, further studies are needed to develop robust, standardized protocols. Bridging these gaps will advance clinical practice and ensure that bariatric patients receive comprehensive, evidence-based care tailored to their unique needs.

## Figures and Tables

**Table 1:** Laboratory markers to evaluate nutritional status.

Marker	Supporting Studies
Urinary creatinine	[68,69,70,71]
Urinary 3-methylhistidine	[72,73,74]
Serum cholesterol	[75,76,77,78]
Delayed hypersensitivity and blood lymphocyte count	[79,80,81,82]
Serum insulin-growth factor 1 (IGF-1)	[83,84,85,86]
Serum Leptin	[87,88,89,90,91,92]
Serum Nesfatin-1	[93,94,95,96]
Serum Zinc	[97,98,99]

**Table 2:** Micronutrient supplementation for bariatric surgery candidates.

Micronutrient	Deficiency Threshold	Recommended Dose	Timing	Notes
Vitamin D [119]	<30 ng/mL (insufficiency)	4000–6000 IU/day (severe deficiency)	Begin at least 10 weeks preoperatively	Maintenance dose: 2000 IU/day post-surgery; monitor to avoid hypervitaminosis.
Vitamin B12 [118,120]	<200 pg/mL (deficiency)	1000 µg IM every 2 weeks	Start immediately upon diagnosis	Oral/sublingual: 500–1000 µg/day for mild to moderate deficiencies.
Iron [120]	Ferritin < 30 ng/mL	100–200 mg/day	Begin at least 8–10 weeks preoperatively	Combine with 500 mg vitamin C for improved absorption; consider IV iron for intolerance to oral administration.
Folate [120]	<3.4 ng/mL	2 mg/day	Start immediately upon diagnosis	Ensure concurrent correction of anemia if present.
Zinc [118]	<70 µg/dL	8–11 mg/day	Adjust based on preoperative levels	Monitor levels post-surgery, particularly in malabsorptive procedures like Roux-en-Y gastric bypass.
Calcium [119]	<8.5 mg/dL	1000–1200 mg/day	Integrate into routine supplementation	Combine with 800–1000 IU vitamin D to support bone health.

## Abbreviations

ASMBS	American Society for Metabolic and Bariatric Surgery.
BMI	Body Mass Index.
BS	Bariatric Surgery.
CRP	C-Reactive Protein.
ESPEN	European Society for Clinical Nutrition and Metabolism.
GLIM	Global Leadership Initiative on Malnutrition.
GNRI	Geriatric Nutrition Risk Index.
IM	Intramuscular.
LCD	Low-Calorie Diet.
MNA	Mini Nutritional Assessment.
MNA-SF	Mini Nutritional Assessment-Short Form.
MST	Malnutrition Screening Tool.
MUST	Malnutrition Universal Screening Tool.
MUS	Non-Alcoholic Fatty Liver Disease.
NRI	Nutritional Risk Index.
PTH	Parathyroid Hormone.
RYGB	Roux-en-Y Gastric Bypass.
SG	Sleeve Gastrectomy.
VLEKT	Very Low Energy Ketogenic Therapy.
VLCKD	Very Low-Calorie Ketogenic Diet.
VLCD	Very Low-Calorie Diet.

## References

[1] World Health Organization Obesity and Overweight. In World Health Organization News. 1 March 2024. https://www.who.int/news-room/fact-sheets/detail/obesity-and-overweight.

[2] Sjöström L., Lindroos A.-K., Peltonen M., Torgerson J., Bouchard C., Carlsson B., Dahlgren S., Larsson B., Narbro K., Sjöström C.D. (2004). Lifestyle, Diabetes, and Cardiovascular Risk Factors 10 Years after Bariatric Surgery. N. Engl. J. Med..

[3] Zarshenas N., Tapsell L.C., Neale E.P., Batterham M., Talbot M.L. (2020). The Relationship Between Bariatric Surgery and Diet Quality: A Systematic Review. Obes. Surg..

[4] Shannon C., Gervasoni A., Williams T. (2013). The bariatric surgery patient—Nutrition considerations. Aust. Fam. Physician.

[5] Aills L., Blankenship J., Buffington C., Furtado M., Parrott J. (2008). ASMBS Allied Health Nutritional Guidelines for the Surgical Weight Loss Patient. Surg. Obes. Relat. Dis..

[6] Buchwald H., Estok R., Fahrbach K., Banel D., Jensen M.D., Pories W.J., Bantle J.P., Sledge I. (2009). Weight and Type 2 Diabetes after Bariatric Surgery: Systematic Review and Meta-analysis. Am. J. Med..

[7] Schiavo L., De Stefano G., Persico F., Gargiulo S., Di Spirito F., Griguolo G., Petrucciani N., Fontas E., Iannelli A., Pilone V. (2021). A Randomized, Controlled Trial Comparing the Impact of a Low-Calorie Ketogenic vs a Standard Low-Calorie Diet on Fat-Free Mass in Patients Receiving an Elipse™ Intragastric Balloon Treatment. Obes. Surg..

[8] Dagan S.S., Goldenshluger A., Globus I., Schweiger C., Kessler Y., Sandbank G.K., Ben-Porat T., Sinai T. (2017). Nutritional Recommendations for Adult Bariatric Surgery Patients: Clinical Practice. Adv. Nutr..

[9] Riess K.P., Baker M.T., Lambert P.J., Mathiason M.A., Kothari S.N. (2008). Effect of preoperative weight loss on laparoscopic gastric bypass outcomes. Surg. Obes. Relat. Dis..

[10] Sarno G., Calabrese P., Frias-Toral E., Ceriani F., Fuchs-Tarlovsky V., Spagnuolo M., Cucalón G., Córdova L.Á., Schiavo L., Pilone V. (2023). The relationship between preoperative weight loss and intra and post-bariatric surgery complications: An appraisal of the current preoperative nutritional strategies. Crit. Rev. Food Sci. Nutr..

[11] Sivakumar J., Chong L., Ward S., Sutherland T.R., Read M., Hii M.W. (2020). Body Composition Changes Following a Very-Low-Calorie Pre-Operative Diet in Patients Undergoing Bariatric Surgery. Obes. Surg..

[12] van Wissen J., Bakker N., Doodeman H.J., Jansma E.P., Bonjer H.J., Houdijk A.P.J. (2016). Preoperative Methods to Reduce Liver Volume in Bariatric Surgery: A Systematic Review. Obes. Surg..

[13] Sarno G., Schiavo L., Calabrese P., Córdova L.Á., Frias-Toral E., Cucalón G., Garcia-Velasquez E., Fuchs-Tarlovsky V., Pilone V. (2022). The Impact of Bariatric-Surgery-Induced Weight Loss on Patients Undergoing Liver Transplant: A Focus on Metabolism, Pathophysiological Changes, and Outcome in Obese Patients Suffering NAFLD-Related Cirrhosis. J. Clin. Med..

[14] Jastrzębska W., Boniecka I., Szostak-Węgierek D. (2021). Validity and efficacy of diets used for preoperative weight reduction among patients qualified for bariatric surgery. Pol. J. Surg..

[15] Mocanu V., Marcil G., Dang J.T., Birch D.W., Switzer N.J., Karmali S. (2021). Preoperative weight loss is linked to improved mortality and leaks following elective bariatric surgery: An analysis of 548,597 patients from 2015–2018. Surg. Obes. Relat. Dis..

[16] Carriel-Mancilla J., Suárez R., Frias-Toral E., Bautista-Valarezo E., Zambrano T.A., García A.A., Jaramillo R.M., Ferrín M., Martin J., Ramos A.C. (2024). Short-medium term complications of bariatric surgery: A pilot study. Minerva Endocrinol..

[17] Cassie S., Menezes C., Birch D.W., Shi X., Karmali S. (2011). Effect of preoperative weight loss in bariatric surgical patients: A systematic review. Surg. Obes. Relat. Dis..

[18] de Sousa J.P.V., Santos-Sousa H., Vieira S., Nunes R., Nogueiro J., Pereira A., Resende F., Costa-Pinho A., Preto J., Sousa-Pinto B. (2024). Assessing Nutritional Deficiencies in Bariatric Surgery Patients: A Comparative Study of Roux-en-Y Gastric Bypass versus Sleeve Gastrectomy. J. Pers. Med..

[19] Al-Maskari J., Al-Hadhrami B., Waly M.I., Al Subhi L., Ali A. (2024). Assessment of dietary intake and biochemical parameters of morbidly obese Omani patients who are candidates for bariatric surgery. Clin. Nutr. Open Sci..

[20] Gazouli A. (2024). Perioperative nutritional assessment and management of patients undergoing gastrointestinal surgery. Ann. Gastroenterol..

[21] Schiavo L., Scalera G., Pilone V., De Sena G., Capuozzo V., Barbarisi A. (2015). Micronutrient Deficiencies in Patients Candidate for Bariatric Surgery: A Prospective, Preoperative Trial of Screening, Diagnosis, and Treatment. Int. J. Vitam. Nutr. Res..

[22] Thieme R.D., Cutchma G., Chieferdecker M.E.M., Campos A.C.L. (2013). O índice de risco nutricional (nutritional risk index) é preditor de complicação pós-operatória em operações do aparelho digestivo ou parede abdominal?. ABCD Arquivos Bras. Cir. Dig..

[23] Ben-Porat T., Elazary R., Yuval J.B., Wieder A., Khalaileh A., Weiss R. (2015). Nutritional deficiencies after sleeve gastrectomy: Can they be predicted preoperatively?. Surg. Obes. Relat. Dis..

[24] Chapela S.P., Martinuzzi A.L.N., Llobera N.D., Ceriani F., Gonzalez V., Montalvan M., Verde L., Frias-Toral E. (2024). Obesity and micronutrients deficit, when and how to suplement. Food Agric. Immunol..

[25] Schiavo L., Pilone V., Rossetti G., Romano M., Pieretti G., Schneck A.-S., Iannelli A. (2019). Correcting micronutrient deficiencies before sleeve gastrectomy may be useful in preventing early postoperative micronutrient deficiencies. Int. J. Vitam. Nutr. Res..

[26] Barrea L., Frias-Toral E., Pugliese G., Garcia-Velasquez E., Carignano M.D.L.A., Savastano S., Colao A., Muscogiuri G. (2021). Vitamin D in obesity and obesity-related diseases: An overview. Minerva Endocrinol..

[27] Flancbaum L., Belsley S., Drake V., Colarusso T., Tayler E. (2006). Preoperative Nutritional Status of Patients Undergoing Roux-en-Y Gastric Bypass for Morbid Obesity. J. Gastrointest. Surg..

[28] Damms-Machado A., Friedrich A., Kramer K.M., Stingel K., Meile T., Küper M.A., Königsrainer A., Bischoff S.C. (2012). Pre- and Postoperative Nutritional Deficiencies in Obese Patients Undergoing Laparoscopic Sleeve Gastrectomy. Obes. Surg..

[29] Pilone V., Tramontano S., Cutolo C., Marchese F., Pagano A.M., Di Spirito F., Schiavo L. (2020). Clinical factors correlated with vitamin D deficiency in patients with obesity scheduled for bariatric surgery: A single center experience. Int. J. Vitam. Nutr. Res..

[30] Stein E.M., Strain G., Sinha N., Ortiz D., Pomp A., Dakin G., McMahon D.J., Bockman R., Silverberg S.J. (2009). Vitamin D insufficiency prior to bariatric surgery: Risk factors and a pilot treatment study. Clin. Endocrinol..

[31] Chakhtoura M.T., Nakhoul N., Akl E.A., Mantzoros C.S., El Hajj Fuleihan G.A. (2016). Guidelines on vitamin D replacement in bariatric surgery: Identification and systematic appraisal. Metabolism.

[32] Iglar P.J., Hogan K.J. (2015). Vitamin D status and surgical outcomes: A systematic review. Patient Saf. Surg..

[33] Gasmi A., Bjørklund G., Mujawdiya P.K., Semenova Y., Peana M., Dosa A., Piscopo S., Benahmed A.G., Costea D.O. (2022). Micronutrients deficiences in patients after bariatric surgery. Eur. J. Nutr..

[34] Moizé V., Deulofeu R., Torres F., de Osaba J.M., Vidal J. (2011). Nutritional Intake and Prevalence of Nutritional Deficiencies Prior to Surgery in a Spanish Morbidly Obese Population. Obes. Surg..

[35] Aasheim E.T., Hofsø D., Hjelmesæth J., Birkeland K.I., Bøhmer T. (2008). Vitamin status in morbidly obese patients: A cross-sectional study. Am. J. Clin. Nutr..

[36] Giuseppina B., Francesca G., Rezarta K., Laura T., Antonella D.M., Alessandra P., Rosaria I.M., Daniela L., Vito D.G.C., Vigna L. (2024). Lifestyle intervention in workers with obesity and sedentary behavior: A pilot study for the “OTTiMo LavorO” project. Mediterr. J. Nutr. Metab..

[37] Muscogiuri G., Barrea L., Frias-Toral E., Garcia-Velasquez E., de Angelis C., Ordoñez C., Cucalón G., El Ghoch M., Colao A., Pivonello R. (2023). Environmental Impact on Metabolism. Environmental Endocrinology and Endocrine Disruptors.

[38] Bayraktaroglu E., Hizli-Guldemir H., Eti S., Kayali-Sevim M., Saleki N. (2024). The relationship between perceived stress and emotional eating in bus drivers: The effect of shift work. Int. J. Food Sci. Nutr..

[39] Frame-Peterson L.A., Megill R.D., Carobrese S., Schweitzer M. (2017). Nutrient Deficiencies Are Common Prior to Bariatric Surgery. Nutr. Clin. Pract..

[40] Grosso G., Laudisio D., Frias-Toral E., Barrea L., Muscogiuri G., Savastano S., Colao A. (2022). Anti-Inflammatory Nutrients and Obesity-Associated Metabolic-Inflammation: State of the Art and Future Direction. Nutrients.

[41] Mohapatra S., Gangadharan K., Pitchumoni C.S. (2020). Malnutrition in obesity before and after bariatric surgery. Dis. Month.

[42] Coupaye M., Rivière P., Breuil M.C., Castel B., Bogard C., Dupré T., Flamant M., Msika S., Ledoux S. (2014). Comparison of Nutritional Status During the First Year After Sleeve Gastrectomy and Roux-en-Y Gastric Bypass. Obes. Surg..

[43] van Rutte P.W.J., Aarts E.O., Smulders J.F., Nienhuijs S.W. (2014). Nutrient Deficiencies Before and After Sleeve Gastrectomy. Obes. Surg..

[44] Schweiger C., Weiss R., Berry E., Keidar A. (2010). Nutritional Deficiencies in Bariatric Surgery Candidates. Obes. Surg..

[45] Al-Mutawa A., Anderson A.K., Alsabah S., Al-Mutawa M. (2018). Nutritional Status of Bariatric Surgery Candidates. Nutrients.

[46] Wellen K.E., Hotamisligil G.S. (2005). Inflammation, stress, and diabetes. J. Clin. Investig..

[47] Marcos J.L., Olivares-Barraza R., Ceballo K., Wastavino M., Ortiz V., Riquelme J., Martínez-Pinto J., Muñoz P., Cruz G., Sotomayor-Zárate R. (2023). Obesogenic Diet-Induced Neuroinflammation: A Pathological Link between Hedonic and Homeostatic Control of Food Intake. Int. J. Mol. Sci..

[48] Schumann R., Ziemann-Gimmel P., Sultana A., Eldawlatly A.A., Kothari S.N., Shah S., Wadhwa A. (2021). Postoperative nausea and vomiting in bariatric surgery: A position statement endorsed by the ASMBS and the ISPCOP. Surg. Obes. Relat. Dis..

[49] Ferraz Á.A.B., Carvalho M.R.C., Siqueira L.T., Santa-Cruz F., Campos J.M. (2018). Deficiências de micronutrientes após cirurgia bariátrica: Análise comparativa entre gastrectomia vertical e derivação gástrica em Y de Roux. Rev. Col. Bras. Cir..

[50] Schiavo L., Aliberti S.M., Calabrese P., Senatore A.M., Severino L., Sarno G., Iannelli A., Pilone V. (2022). Changes in Food Choice, Taste, Desire, and Enjoyment 1 Year after Sleeve Gastrectomy: A Prospective Study. Nutrients.

[51] Barrea L., Verde L., Schiavo L., Sarno G., Camajani E., Iannelli A., Caprio M., Pilone V., Colao A., Muscogiuri G. (2023). Very Low-Calorie Ketogenic Diet (VLCKD) as Pre-Operative First-Line Dietary Therapy in Patients with Obesity Who Are Candidates for Bariatric Surgery. Nutrients.

[52] Bettini S., Belligoli A., Fabris R., Busetto L. (2020). Diet approach before and after bariatric surgery. Rev. Endocr. Metab. Disord..

[53] White J.V., Guenter P., Jensen G., Malone A., Schofield M. (2012). Consensus Statement: Academy of Nutrition and Dietetics and American Society for Parenteral and Enteral Nutrition. J. Parenter. Enter. Nutr..

[54] Parrott J., Frank L., Rabena R., Craggs-Dino L., Isom K.A., Greiman L. (2017). American Society for Metabolic and Bariatric Surgery Integrated Health Nutritional Guidelines for the Surgical Weight Loss Patient 2016 Update: Micronutrients. Surg. Obes. Relat. Dis..

[55] Williams D.G., Molinger J., Wischmeyer P.E. (2019). The malnourished surgery patient. Curr. Opin. Anaesthesiol..

[56] Sherf-Dagan S., Sinai T., Goldenshluger A., Globus I., Kessler Y., Schweiger C., Ben-Porat T. (2021). Nutritional Assessment and Preparation for Adult Bariatric Surgery Candidates: Clinical Practice. Adv. Nutr. Int. Rev. J..

[57] Keller U. (2019). Nutritional Laboratory Markers in Malnutrition. J. Clin. Med..

[58] Sproston N.R., Ashworth J.J. (2018). Role of C-Reactive Protein at Sites of Inflammation and Infection. Front. Immunol..

[59] Gonzales G.F., Moreno V.J.S. (2024). Niveles de hemoglobina para la determinación de la anemia: Nueva guía de la Organización Mundial de la Salud y adecuación de la norma nacional. Rev. Peru Med. Exp. Salud Publica.

[60] Kuzuya M., Kanda S., Koike T., Suzuki Y., Iguchi A. (2005). Lack of correlation between total lymphocyte count and nutritional status in the elderly. Clin. Nutr..

[61] Cabrerizo S., Cuadras D., Gomez-Busto F., Artaza-Artabe I., Marín-Ciancas F., Malafarina V. (2015). Serum albumin and health in older people: Review and meta analysis. Maturitas.

[62] Kudsk K., Tolley E., DeWitt R., Janu P., Blackwell A., Yeary S., King B. (2003). Preoperative albumin and surgical site identify surgical risk for major postoperative complications. J. Parenter. Enter. Nutr..

[63] Dellière S., Cynober L. (2016). Is transthyretin a good marker of nutritional status?. Clin. Nutr..

[64] Bharadwaj S., Ginoya S., Tandon P., Gohel T.D., Guirguis J., Vallabh H., Jevenn A., Hanouneh I. (2016). Malnutrition: Laboratory markers vs nutritional assessment. Gastroenterol. Rep..

[65] Sergi G., Coin A., Enzi G., Volpato S., Inelmen E.M., Buttarello M., Peloso M., Mulone S., Marin S., Bonometto P. (2006). Role of visceral proteins in detecting malnutrition in the elderly. Eur. J. Clin. Nutr..

[66] Zhang Z., Pereira S.L., Luo M., Matheson E.M. (2017). Evaluation of Blood Biomarkers Associated with Risk of Malnutrition in Older Adults: A Systematic Review and Meta-Analysis. Nutrients.

[67] Ingenbleek Y. (2019). Plasma Transthyretin as A Biomarker of Sarcopenia in Elderly Subjects. Nutrients.

[68] David D., Poli C., Savian J., Amaral G., Azevedo E., Jochims F. (2015). Urinary creatinine as a nutritional and urinary volume marker in sheep fed with tropical or temperate forages. Arq. Bras. Med. Vet. Zootec..

[69] Remer T., Neubert A., Maser-Gluth C. (2002). Anthropometry-based reference values for 24-h urinary creatinine excretion during growth and their use in endocrine and nutritional research. Am. J. Clin. Nutr..

[70] Malinowska-Borowska J., Kulik A., Buczkowska M., Ostręga W., Stefaniak A., Piecuch M., Garbicz J., Nowak J.U., Tajstra M., Jankowska E.A. (2021). Nutritional and Non-Nutritional Predictors of Low Spot Urinary Creatinine Concentration in Patients with Heart Failure. Nutrients.

[71] Adewusi S., Torimiro S., Akindahunsi A. (2002). Prediction of Nutritional Status by Chemical Analysis of Urine and Anthropometric Methods. Nutr. Health.

[72] Hoffer L. (1990). Nutritional status affects renal 3-methylhistidine handling in humans. Metabolism.

[73] Long C.L., Birkhahn R.H., Geiger J.W., Betts J.E., Schiller W.R., Blakemore W.S. (1981). Urinary excretion of 3-methylhistidine: An assessment of muscle protein catabolism in adult normal subjects and during malnutrition, sepsis, and skeletal trauma. Metabolism.

[74] Yamada T., Kurasawa S.-I., Matsuzaki M., Tanaka A. (2023). Body weight reduction by exercise increases the urinary 3-methylhistidine excretion level with relatively positive nitrogen, sodium, and potassium balances when compared to dietary restriction. Heliyon.

[75] Jawzali J., Saber H., Khalil K. (2017). Assessment of nutritional status among dyslipidemia patients. Saudi J. Health Sci..

[76] Delisle H., Ntandou G., Sodjinou R., Couillard C., Després J.-P. (2013). At-Risk Serum Cholesterol Profile at Both Ends of the Nutrition Spectrum in West African Adults? The Benin Study. Nutrients.

[77] Araújo J.P., Friões F., Azevedo A., Lourenço P., Rocha-Gonçalves F., Ferreira A., Bettencourt P. (2008). Cholesterol—A marker of nutritional status in mild to moderate heart failure. Int. J. Cardiol..

[78] Itoh K., Imai K., Masuda T., Abe S., Nakao H., Tanaka M., Nakamura M. (1999). Relationship between serum total cholesterol level and nutritional status in Japanese young female. Nutr. Res..

[79] Sue M., Takeuchi Y., Hirata S., Takaki A., Otsuka M. (2024). Impact of Nutritional Status on Neutrophil-to-Lymphocyte Ratio as a Predictor of Efficacy and Adverse Events of Immune Check-Point Inhibitors. Cancers.

[80] Dionigi P., Jemos V., Cebrelli T. (1992). Serum and Immunological Parameters in the Assessment of Nutritional Status. Nutrition and Ventilatory Function.

[81] Dionigi R. (1982). Immunological factors in nutritional assessment. Proc. Nutr. Soc..

[82] Usta M., Ersoy A., Ayar Y., Budak F. (2020). The relationship between lymphocyte subsets, nutritional status and tuberculin reactivity in continuous ambulatory peritoneal dialysis and hemodialysis patients. Int. Urol. Nephrol..

[83] Wang X., Tian F., Sun H., Zhang L., Gao X., Huang Y., Yang J., Shen R., Wang J., Jiang T. (2019). Insulin-like growth factor-1 as a nutritional monitoring factor in patients with chronic intestinal failure. Clin. Nutr..

[84] Fouad H.M., Mohamed A.A., Adel N., Abdulhay M., Khalifa I., Ibrahim R., Elsalway N., Thabet G.M., Nasraldin K., El-Hefny I.M. (2024). Evaluation of insulin-like growth factor-1 in apparently healthy infants and prepubertal Egyptian children with different nutritional statuses. BMC Pediatr..

[85] Caregaro L., Favaro A., Santonastaso P., Alberino F., DI Pascoli L., Nardi M., Favaro S., Gatta A. (2001). Insulin-like growth factor 1 (IGF-1), a nutritional marker in patients with eating disorders. Clin. Nutr..

[86] Hawkes C.P., Grimberg A. (2015). Insulin-Like Growth Factor-I is a Marker for the Nutritional State. Pediatr. Endocrinol. Rev..

[87] Amarase C., Weerasopone S., Osateerakun P., Honsawek S., Limpaphayom N. (2016). Serum Leptin as a Nutritional Biomarker in Children with Cerebral Palsy. Tohoku J. Exp. Med..

[88] Ma W., Zheng Y., Lin J., Zhou S., Liao S., Fu Y., Zhang Y., Chen X., Li J., Sha W. (2023). Circulating leptin levels in the assessment of Crohn’s disease activity and its relation to nutritional status. Nutr. Hosp..

[89] Ko Y., Lin Y., Kuo C., Lai Y., Wang C., Hsu B. (2020). Low serum leptin levels are associated with malnutrition status according to malnutrition-inflammation score in patients undergoing chronic hemodialysis. Hemodial. Int..

[90] Oztekin M.G., Erel S., Kismet K., Kilicoglu B., Gencay C., Astarci H.M., Akkus M.A. (2007). Use of serum leptin levels for determination of nutritional status and the effects of different enteral nutrients on intestinal mucosa after minor surgery: An experimental study. Int. J. Surg..

[91] Akib R.D., Aminuddin A., Hamid F., Prihantono P., Bahar B., Hadju V. (2021). Leptin levels in children with malnutrition. Gac. Sanit..

[92] Paillaud E., Poisson J., Granier C., Ginguay A., Plonquet A., Conti C., Broussier A., Raynaud-Simon A., Bastuji-Garin S. (2022). Serum Leptin Levels, Nutritional Status, and the Risk of Healthcare-Associated Infections in Hospitalized Older Adults. Nutrients.

[93] Samani S. (2019). Serum Nesfatin-1 Level in Healthy Subjects with Weight-Related Abnormalities and Newly Diagnosed Patients with Type 2 Diabetes Mellitus; a Case-Control Study. Acta Endocrinol..

[94] Çoban Y., Köker A., Aydın S., Akbaş Y., Kömüroğlu A.U. (2023). Investigation of the Role of Nesfatin-1 Levels in the Evaluation of Nutrition Monitoring in the PICU. Turk. J. Pediatr. Emerg. Intensiv. Care Med..

[95] Stengel A., Mori M., Taché Y. (2013). The role of nesfatin-1 in the regulation of food intake and body weight: Recent developments and future endeavors. Obes. Rev..

[96] Elthakaby A.H.M., Elsayed H.M., Mohamed N.A.E.-G., Ahmed K.Y., Mohamed R.R., Esso H.Y. (2022). Effect of nesfatin-1 on the nutritional status of hemodialysis patients. J. Med. Sci. Res..

[97] Ahsan A.K., Tebha S.S., Sangi R., Kamran A., Zaidi Z.A., Haque T., Hamza M.S.A. (2021). Zinc Micronutrient Deficiency and Its Prevalence in Malnourished Pediatric Children as Compared to Well-Nourished Children: A Nutritional Emergency. Glob. Pediatr. Health.

[98] Özen H., Emiroğlu H.H., Emiroğlu M., Akdam N., Yorulmaz A. (2023). Serum Zinc in Patients with Protein-Energy Malnutrition Retrospective Assessment of Levels. Genel Tıp Dergisi..

[99] Abeywickrama H.M., Uchiyama M., Sumiyoshi T., Okuda A., Koyama Y. (2024). The role of zinc on nutritional status, sarcopenia, and frailty in older adults: A scoping review. Nutr. Rev..

[100] Toh S.Y., Zarshenas N., Jorgensen J. (2009). Prevalence of nutrient deficiencies in bariatric patients. Nutrition.

[101] Fuhrman M. (2002). The Albumin-nutrition connection: Separating myth from fact. Nutrition.

[102] Kushiyama S., Sakurai K., Kubo N., Tamamori Y., Nishii T., Tachimori A., Inoue T., Maeda K. (2018). The Preoperative Geriatric Nutritional Risk Index Predicts Postoperative Complications in Elderly Patients with Gastric Cancer Undergoing Gastrectomy. In Vivo.

[103] Buzby G.P., Mullen J.L., Matthews D.C., Hobbs C.L., Rosato E.F. (1980). Prognostic nutritional index in gastrointestinal surgery. Am. J. Surg..

[104] Oh C.A. (2012). Nutritional risk index as a predictor of postoperative wound complications after gastrectomy. World J. Gastroenterol..

[105] Bouillanne O., Morineau G., Dupont C., Coulombel I., Vincent J.-P., Nicolis I., Benazeth S., Cynober L., Aussel C. (2005). Geriatric Nutritional Risk Index: A new index for evaluating at-risk elderly medical patients. Am. J. Clin. Nutr..

[106] Ferguson M., Capra S., Bauer J., Banks M. (1999). Development of a valid and reliable malnutrition screening tool for adult acute hospital patients. Nutrition.

[107] dos Santos T.A., Luft V.C., Souza G.C., Santos Z.d.A., Jochims A.M.K., de Almeida J.C. (2023). Malnutrition screening tool and malnutrition universal screening tool as a predictors of prolonged hospital stay and hospital mortality: A cohort study. Clin. Nutr. ESPEN.

[108] Stratton R.J., Hackston A., Longmore D., Dixon R., Price S., Stroud M., King C., Elia M. (2004). Malnutrition in hospital outpatients and inpatients: Prevalence, concurrent validity and ease of use of the ‘malnutrition universal screening tool’ (‘MUST’) for adults. Br. J. Nutr..

[109] Almasaudi A.S., McSorley S.T., Dolan R.D., Edwards C.A., McMillan D.C. (2019). The relation between Malnutrition Universal Screening Tool (MUST), computed tomography–derived body composition, systemic inflammation, and clinical outcomes in patients undergoing surgery for colorectal cancer. Am. J. Clin. Nutr..

[110] Serón-Arbeloa C., Labarta-Monzón L., Puzo-Foncillas J., Mallor-Bonet T., Lafita-López A., Bueno-Vidales N., Montoro-Huguet M. (2022). Malnutrition Screening and Assessment. Nutrients.

[111] Kondrup J., Rasmussen H.H., Hamberg O., Stanga Z. (2003). Nutritional risk screening (NRS 2002): A new method based on an analysis of controlled clinical trials. Clin. Nutr..

[112] Yıldırım R., Candaş B., Usta M.A., Erkul O., Türkyılmaz S., Güner A. (2020). Comparison of Nutritional Screening Tools in Patients Undergoing Surgery for Gastric Cancer. Med. Bull. Haseki.

[113] Rubenstein L.Z., Harker J.O., Salvà A., Guigoz Y., Vellas B. (2001). Screening for Undernutrition in Geriatric Practice: Developing the Short-Form Mini-Nutritional Assessment (MNA-SF). J. Gerontol. Ser. A.

[114] Soysal P., Veronese N., Arik F., Kalan U., Smith L., Isik A.T. (2019). Mini Nutritional Assessment Scale-Short Form can be useful for frailty screening in older adults. Clin. Interv. Aging.

[115] Detsky A.S., McLaughlin J.R., Baker J.P., Johnston N., Whittaker S., Mendelson R.A., Jeejeebhoy K.N. (1987). What is subjective global assessment of nutritional status?. J. Parenter. Enter. Nutr..

[116] Pham N., Coxreijven P., Greve J., Soeters P. (2006). Application of subjective global assessment as a screening tool for malnutrition in surgical patients in Vietnam. Clin. Nutr..

[117] Fink J.d.S., de Mello P.D., de Mello E.D. (2015). Subjective global assessment of nutritional status—A systematic review of the literature. Clin. Nutr..

[118] Ukleja A., Gilbert K., Mogensen K.M., Walker R., Ward C.T., Ybarra J., Holcombe B., Ukleja A.A., Gilbert M.K., Mogensen R.K.M. (2018). Standards for Nutrition Support: Adult Hospitalized Patients. Nutr. Clin. Pract..

[119] Cederholm T., Barazzoni R., Austin P., Ballmer P., Biolo G., Bischoff S.C., Compher C., Correia I., Higashiguchi T., Holst M. (2017). ESPEN guidelines on definitions and terminology of clinical nutrition. Clin. Nutr..

[120] Schiavo L., Scalera G., Sergio R., De Sena G., Pilone V., Barbarisi A. (2015). Clinical impact of Mediterranean-enriched-protein diet on liver size, visceral fat, fat mass, and fat-free mass in patients undergoing sleeve gastrectomy. Surg. Obes. Relat. Dis..

[121] Schiavo L., Pilone V., Rossetti G., Barbarisi A., Cesaretti M., Iannelli A. (2018). A 4-Week Preoperative Ketogenic Micronutrient-Enriched Diet Is Effective in Reducing Body Weight, Left Hepatic Lobe Volume, and Micronutrient Deficiencies in Patients Undergoing Bariatric Surgery: A Prospective Pilot Study. Obes. Surg..

[122] Pilone V., Tramontano S., Renzulli M., Romano M., Cobellis L., Berselli T., Schiavo L. (2018). Metabolic effects, safety, and acceptability of very low-calorie ketogenic dietetic scheme on candidates for bariatric surgery. Surg. Obes. Relat. Dis..

[123] Suarez R., Chapela S., Llobera N.D., Montalván M., Vásquez C.A., Martinuzzi A.L.N., Katsanos C.S., Verde L., Frias-Toral E., Barrea L. (2024). Very Low Calorie Ketogenic Diet: What Effects on Lipid Metabolism?. Curr. Nutr. Rep..

[124] Barrea L., Caprio M., Grassi D., Cicero A.F.G., Bagnato C., Paolini B., Muscogiuri G. (2024). A New Nomenclature for the Very Low-Calorie Ketogenic Diet (VLCKD): Very Low-Energy Ketogenic Therapy (VLEKT). Ketodiets and Nutraceuticals Expert Panels: “KetoNut”, Italian Society of Nutraceuticals (SINut) and the Italian Association of Dietetics and Clinical Nutrition (ADI). Curr. Nutr. Rep..

[125] Castaldo G., Schiavo L., Pagano I., Molettieri P., Conte A., Sarno G., Pilone V., Rastrelli L. (2023). Clinical Impact of Enteral Protein Nutritional Therapy on Patients with Obesity Scheduled for Bariatric Surgery: A Focus on Safety, Efficacy, and Pathophysiological Changes. Nutrients.

[126] Berardi G., Vitiello A., Abu-Abeid A., Schiavone V., Franzese A., Velotti N., Musella M. (2023). Micronutrients Deficiencies in Candidates of Bariatric Surgery: Results from a Single Institution over a 1-Year Period. Obes. Surg..

[127] Giustina A., di Filippo L., Facciorusso A., Adler R.A., Binkley N., Bollerslev J., Bouillon R., Casanueva F.F., Cavestro G.M., Chakhtoura M. (2023). Vitamin D status and supplementation before and after Bariatric Surgery: Recommendations based on a systematic review and meta-analysis. Rev. Endocr. Metab. Disord..

[128] O’Kane M., Parretti H.M., Pinkney J., Welbourn R., Hughes C.A., Mok J., Walker N., Thomas D., Devin J., Coulman K.D. (2020). British Obesity and Metabolic Surgery Society Guidelines on perioperative and postoperative biochemical monitoring and micronutrient replacement for patients undergoing bariatric surgery—2020 update. Obes. Rev..

[129] Leahy C.R., Luning A. (2015). Review of Nutritional Guidelines for Patients Undergoing Bariatric Surgery. AORN J..

[130] Barrea L., Salzano C., Pugliese G., Laudisio D., Frias-Toral E., Savastano S., Colao A., Muscogiuri G. (2022). The challenge of weight loss maintenance in obesity: A review of the evidence on the best strategies available. Int. J. Food Sci. Nutr..

[131] Schiavo L., Pilone V., Rossetti G., Iannelli A. (2019). The Role of the Nutritionist in a Multidisciplinary Bariatric Surgery Team. Obes. Surg..

[132] Verde L., Frias-Toral E., Cardenas D. (2023). Editorial: Environmental factors implicated in obesity. Front. Nutr..

[133] Chaim E.A., Pareja J.C., Gestic M.A., Utrini M.P., Cazzo E. (2017). Preoperative multidisciplinary program for bariatric surgery: A proposal for the Brazilian Public Health System. Arq. Gastroenterol..

[134] Thompson R., Farrell T.M. (2020). Importance of a Multidisciplinary Approach for Bariatric Surgery. Foregut Surgery.

[135] Patel P., Hartland A., Hollis A., Ali R., Elshaw A., Jain S., Khan A., Mirza S. (2015). Tier 3 multidisciplinary medical weight management improves outcome of Roux-en-Y gastric bypass surgery. Ind. Mark. Manag..

[136] Delgado Floody P., Caamaño Navarrete F., Jerez Mayorga D., Campos Jara C., Ramírez Campillo R., Osorio Poblete A., Hormazábal M.A., Lepeley N.T., Mansilla C.S. (2015). Effects of a multidisciplinary program on morbid obese patients and patients with comorbility who are likely to be candidates for bariatric surgery. Nutr. Hosp..

[137] Gorodner V., Di Corpo M., Schlottmann F. (2020). Tailoring Surgical Treatment for the Individual Patient. Foregut Surgery.

[138] Tursun S., Şahin Y., Alçiğir M.E., Çínar M., Karahan I. (2024). Cafeteria diet can cause systemic inflammation and oxidative damage in the various tissues. Mediterr. J. Nutr. Metab..

[139] Golshany H., Helmy S.A., Morsy N.F.S., Kamal A., Yu Q., Fan L. (2024). The gut microbiome across the lifespan: How diet modulates our microbial ecosystem from infancy to the elderly. Int. J. Food Sci. Nutr..

[140] Obeid N.R., Dimick J.B. (2020). Sleeve Gastrectomy. Foregut Surgery.

[141] Laxague F., Schlottmann F., Buxhoeveden R. (2020). Laparoscopic Roux-en-Y Gastric Bypass. Foregut Surgery.

